# Teaching clinical phenomenology in a post-graduate medical training program

**DOI:** 10.1192/j.eurpsy.2024.578

**Published:** 2024-08-27

**Authors:** L. Mehl-Madrona

**Affiliations:** Native Studies, University of Maine, Orono, United States

## Abstract

**Introduction:**

Patient care suffers when practitioners do not understand the patient’s experience of the illness. This is especially true in psychiatry relative to patients having learned the jargon and studying the diagnostic criteria for various mental disorders using internet search engines, especially Google. Patients more commonly present with a list of symptoms that match the checklist for their self-diagnosis. Their experience may be quite different from the words they use to present their symptoms to medical personnel. More than ever, psychiatric providers need to unpack the words their patients use to discover the actual experience. K. Toombs has written exquisitely about clinical phenomenology in the context of her diagnosis of multiple sclerosis and her negotiation of a medical system that largely ignored her lived experience. This algorithmic, checklist approach to human suffering derives from an understanding of clinical practice in which the practitioner applies scientific knowledge of the bodily processes of diseases, recognizing those diseases by inquiring about the presence of symptoms characterizing those diseases and then rendering treatments based upon statistical studies (preferably randomized, controlled trials) of large groups of individuals. Within this approach, little need exists to encounter the person with the disease as an actual human being, an Other in the sense of Levinas. This approach suffers because even history-taking requires an encounter with an Other in which the internal, private experience of this Other must be heard and made part of the clinical process. This approach can ignore the role of the doctor-patient relationship and the clinical encounter in symptom relief.

**Objectives:**

To describe an educational program in clinical phenomenology that was well received by trainees who rated that it improved their practice.

**Methods:**

In this presentation, we will describe our efforts at teaching clinical phenomenology and psychiatry within a residency training program, in which we encourage trainees to develop deeper listening skills through conducting life story interviews, motivational interviewing techniques, and narrative approaches in which they search for the metaphors underlying the patients’ illness. We will describe factors influencing trainees’ acceptance or rejection of these approaches and the change in the culture of our training program and clinic that have arisen from their implementation.

**Results:**

One hundred and twenty residents have taken this course. Eighty-eight percent reported improved clinical skills and ability to relate to patients.

**Conclusions:**

The practice of medicine does not exist independently from the relationships in which medicine is practiced and a thorough understanding of the lived experience and experience of the patient is necessary for accurate diagnosis, psychiatric or medical, and for discovery of a treatment approach with the patient.

**Disclosure of Interest:**

None Declared

